# Cholera Infantum

**Published:** 1871-09

**Authors:** Joel Branham

**Affiliations:** Atlanta, Ga.


### CHOLERA INFANTUM.

BY JOEL BRANHAM, M. D., ATLANTA, GA.

By your request I herewith furnish a brief article on Cholera Infantum—a disease, the treatment of which has troubled the Profession, both in Europe and in this country. I hold that it is the duty of each individual member of the Profession to communicate the results of his experience and practice, especially when such results tend to alleviate the ills of life, or in any way conduce to its preservation. We are often deterred from thus recording our experience by the fear of seeming egotistical so that our literature often merges into fulsome writing, or into a sort of learned profundity that is only intended for show, but never condesends itself to compare the results of different modes of treatment. Our studies are of use only as they make us capable of relieving human suffering. In offering some practical thoughts on the causes and, I think, correct treament of Cholera Infantum, or what is called summer complaint of children, I make no claim to original or profound investigation. The arrangement and suggestions are my own. I have simply attempted to condense a few thoughts which are of vital importance to a proper understanding of the character and treatment of this fatal form of disease. It is not my purpose to write a book or a long article, but call the attention, especially, of the young practitioner—it per chance, may prove interesting to the older members of the Profession who, in this fast age, have not the liesure or inclination to read or study much.

While the process of Dentition is going on, the Sublinqual and Salivary glands are stimulated to over and excessive secretions ; large quantities of saliva are thrown out and swallowed by the child. This, with the two frequent multiplication of articles of food, causes fermentation, and in unnatural quantities is found in the stomach or alimentary canal. Irritation and often inflammation of the mucous membrane is soon established. Another and often a fruitful cause, is the diseased or impure milk of the mother. Many constitutional diseases of the mother, as is well known to all medical men, often does, and will produce this result. Any form of, and disease impairing the general health of the mother, altering the general secretions and causing debility for any length of time, will disease the child, the mother's milk thus becomes unfit food for the child. A want of cleanliness, damp localities, improper clothing, premature exposures in sudden vicisitudes of the mother; many are the causes of this disease, which it is useless here to mention in detail, which are known to mothers and physicians.

Timely medical attention, with proper care on the part of the mother, will often save the little sufferer from a protracted, if not a fatal, spell. The treatment I have pursued for many years is very simple, and within the reach of all. As soon as it is known that the child is teething, the gums should be incised or cut down to the teeth, and repeated as often as may be necessary. The stools are not so frequent at first, but as the disease progresses become thin, offensive and frequent, gradually exhausting and wearing down the child. Often sick stomach and vomiting accompanies this condition of the bowels. As soon as the disease is developed give the following prescription:

| | | |
|---|---|---|
| R.—Calomel, | . . . . . | grs. 3 |
| Prepared Chalk, | . . . . | grs. 12 |
| Pulv. Cinnamon, | . . . . | grs. 6—Mix. |

Make 3 powders, give one every 6 hours in syrup. If the powders should not, in 8 or 10 hours after the last is given, procure 1 or 2 billious stools, a desert spoonful of castor oil may be given.

The quantity of medicine in the above prescription is suited to a child from 1 to 3 years old, for younger children the dose will be lessened by the prudent medical attendant. This prescription may be repeated every 4 or 5 days, or oftener if the frequent thin fœtid stools should continue.

It is now a well-established fact that sulphurous acid and its salts will arrest fermentation. The sulphites, many yeaas ago, were regarded as valuable, but had well-nigh become obsolete; recently this class of remedies have been revived and brought into extensive use with the German and French physicians, and are now being used by them in almost all fever form of disease and have acquired extensive reputation, and are justly regarded as the best correctives known to the medical world. I regard it a great misfortune that this class of medicines for many years, were abandoned. While acidity of the stomach continues or is troublesome, a few grains of the sulphite of soda may be given, in a little mucillage of gum arabic, 4 or 5 times through the day. Opium, or any of its preparations, should be given with great care and caution to infant children, because they are easily impressed, and opium and its preparations does often excite the brain and nervous system to a fatal extent in children. I have often thought that it has been a misfortune to the human family that morphine had ever been discovered. I freely admit it to be a great boon when cautiously and prudently administered. Yet it is a well known and admitted truth that hundreds and thousands have been injured, if not killed, by its indiscriminate and ill-timed administration. A little paragoric is the best preparation of opium for children, and may be given when the child is griped in pain or does not rest well. Children need in this disease a great deal of fluid. I make it an invariable rule to allow the little patient to drink as much pure cold water early in the morning, as it will.

The diet in this disease is of paramount importance. With a patient and persevering attention to the diet, nine cases out of ten will get well. I desire to be particular and well understood by all who may read this article. The food should be sufficiently nourishing and of easy digestion. The best food I have found suited to the disease, is rice and sweet milk, prepared as follows: Boil a sufficient quantity of rice in clear water until it is well done, then add half the quantity of new milk, say half a pint of milk to a pint of the rice and water, boil for half an hour, mash the grains of rice and strain through muslin or gauze, and add a little loaf sugar and nutmeg, confine the child exclusively to this diet. The little one will often resist and refuse, but starve him and in a short time the patient will soon become fond of it. Nothing but a stern determination on the part of the mother or nurse will accomplish this most important end. Keep a constant watch on the character of the stools, and repeat the calomel powders if necessary. If the child should rapidly decline and the rice and milk should not be sufficiently nourishing, as is sometimes the case, raw meat will be found to answer the purpose. Beef is the best. Beat it well. It should be freed from all fibrous and fatty particles, a little salt or sugar may be added, and quince jelly mixed with it. The child will soon like it prepared in this way. This disease often reduces the child until it is greatly emaciated, and needs a mild tonic treatment, after the diseased is checked. Nothing is better than minute does of the sulphate of iron, (pure chrystallized copperas) from a tenth to a sixth or half grain in sweetened water, three times a day. This article sometimes sickens the stomach. Two or three grains of the Sub-Nitrate of Bismuth may be substituted. Proper attention should be paid to the entire surface of the child's body, wash the entire body every 2 or 3 days with tepid soap suds, and as often, gently sponge the body with common whisky or brandy. In extreme cases a blister, the size of a-half dollar, behind each ear has often produced good results. Where there is excessive inflammation of the bowels a blister over the abdomen, not to remain on long enough to make a deep blister, is a remedy I have often used with remarkable benefit. I know it is dangerous and generally a bad practice to blister children, but there does sometimes occur cases where life may and is preserved by their application. I have not attempted any display in this article, but have endeavored to bring to the notice of the Profession a few brief, and I hope some practical thoughts in the management of this disease, so common in the South and so fatal to thousands of children. After 45 years of hard labor in the practice of my much loved and cherished Profession, I thought if I could contribute anything of worth or value to my Professional Brethren, I would be amply rewarded.

